# Burden of New and Recurrent Tuberculosis in a Major South African City Stratified by Age and HIV-Status

**DOI:** 10.1371/journal.pone.0025098

**Published:** 2011-10-10

**Authors:** Robin Wood, Stephen D. Lawn, Judy Caldwell, Richard Kaplan, Keren Middelkoop, Linda-Gail Bekker

**Affiliations:** 1 Desmond Tutu HIV Centre, Institute of Infectious Diseases and Molecular Medicine, University of Cape Town, Cape Town, South Africa; 2 Department of Medicine, University of Cape Town Faculty of Health Sciences, Cape Town, South Africa; 3 Department of Science and Technology/National Research Foundation, Centre of Excellence in Epidemiological Modeling and Analysis, University of Stellenbosch, Cape Town, South Africa; 4 Department of Clinical Research, Faculty of Infectious and Tropical Diseases, London School of Hygiene and Tropical Medicine, London, United Kingdom; 5 City of Cape Town Department of Health, Cape Town, South Africa; San Francisco General Hospital, University of California San Francisco, United States of America

## Abstract

**Aim:**

To describe the burden of tuberculosis (TB) in Cape Town by calculating TB incidence rates stratified by age and HIV-status, assessing the contribution of retreatment disease and estimating the cumulative lifetime TB risk in HIV-negative individuals.

**Methods:**

Details of TB cases were abstracted from the 2009 electronic TB register. Population denominators were estimated from census data and actuarial estimates of HIV prevalence, allowing calculation of age-specific and HIV-stratified TB notification rates.

**Results:**

The 2009 mid-year population was 3,443,010 (3,241,508 HIV-negative and 201,502 HIV-positive individuals). There were 29,478 newly notified TB cases of which 56% were laboratory confirmed. HIV status was recorded for 87% of cases and of those with known HIV-status 49% were HIV-negative and 51% were positive. Discrete peaks in the incidence of non-HIV-associated TB occurred at three ages: 511/100,000 at 0–4 years of age, 553/100,000 at 20–24 years and 628/100,000 at 45–49 years with 1.5%, 19% and 45% being due to retreatment TB, respectively. Only 15.5% of recurrent cases had a history of TB treatment failure or default. The cumulative lifetime risks in the HIV-negative population of all new TB episodes and new smear-positive TB episodes were 24% and 12%, respectively; the lifetime risk of retreatment disease was 9%. The HIV-positive notification rate was 6,567/100,000 (HIV-associated TB rate ratio = 17). Although retreatment cases comprised 30% of the HIV-associated TB burden, 88% of these patients had no history of prior treatment failure or default.

**Conclusions:**

The annual burden of TB in this city is huge. TB in the HIV-negative population contributed almost half of the overall disease burden and cumulative lifetime risks were similar to those reported in the pre-chemotherapy era. Retreatment TB contributed significantly to both HIV-associated and non-HIV-associated TB but infrequently followed prior inadequate treatment. This likely reflects ongoing TB transmission to both HIV-negative and positive individuals.

## Introduction

Tuberculosis (TB) notifications in South Africa have increased progressively over the last 20 years, temporally associated with the growth of the HIV epidemic. South Africa is now the 3^rd^ highest TB burdened country in the world [1], [2] and is estimated to account for approximately 25% of the estimated global caseload of HIV-associated TB [1], [2]. However, testing for HIV-infection among TB cases has remained low due to stigma, doctor reluctance and operational constraints. The prevalence of HIV-infection in South African TB cases in 2007 was estimated to be 74%, but this figure was extrapolated from a minority of notified cases who were tested in that year, potentially leading to over-estimation of HIV prevalence [1], [2]. In South Africa in 2007 HIV-testing was performed on 39% of TB cases with 65% reported positive. However, in 2008 when HIV-testing was increased to 49% of TB cases, 58% were reported positive, resulting in a downward re-estimation of national HIV/TB caseload. Low HIV-testing rates among TB cases therefore constitute a major impediment to our understanding of the epidemiological interaction between TB and HIV at a population level.

Cape Town is a major South African city with a population of 3.4 million people, in which the burden of both HIV and TB are high [3], [4]. The city TB control program is based on a network of community clinics dispensing rifampicin-based TB treatment under a 100% directly observed treatment short course strategy (DOTS) strategy [5], [6] and supported by a comprehensive accredited TB laboratory service [7]. In recent years, there has been a concerted effort to increase provider initiated HIV testing within the TB service [8], [9]. In 2009, HIV-status was determined for 87% of all TB notifications, enabling stratification of the majority of TB cases by HIV-infection status.

We therefore set out to estimate the 2009 age specific TB notification rates for HIV-infected and HIV-uninfected populations of the city. TB numerators were abstracted from the 2009 TB notification database and denominators were estimated from population census data [10], [11] and actuarial estimates of HIV prevalence [12], [13]. Age-specific TB rates were used to define the contribution of new and retreatment TB at different ages and to model cumulative risks for new and retreatment TB.

## Methods

### Population Denominators

The mid-2009 population of Cape Town was estimated using the National Department of Health/Health Information System Program, which disaggregates estimates from Statistics South Africa district using the `Small Area Spacial Laye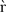 (StatsSA, 2004) of the 2001 national census [10], [11].

Age-specific HIV prevalence was calculated for the City of Cape Town from the province specific output (Western Cape) of the ASSA2003 AIDS and demographic model, which is the current version released by the Actuarial Society of South Africa [12]. The model represents the HIV/AIDS epidemic and its demographic impact on the population of South Africa. Population numbers and age- and sex-stratified HIV prevalence were used to calculate the denominators needed to calculate TB notification rates stratified by age, sex and HIV status.

### Tuberculosis case definitions

TB case definitions were recorded in the electronic TB register as per the guidelines for the National TB Control Program as follows [5]. Transfer in: a case transferred from outside the metropolitan area. Moved in: a case transferred from a facility within the metropolitan area. New case: a patient who has never had treatment for TB or who has taken anti-tuberculosis drugs for less than four weeks. Re-treatment case: a patient who has taken treatment for TB before and either relapsed, defaulted or had treatment failure. Treatment after failure: a pulmonary TB patient who is still sputum smear positive at the end of the treatment period. Treatment after default: a patient who completed at least one month of treatment and returns after having interrupted treatment for two months or more but still with active TB as judged on clinical and radiological assessment. Smear positive pulmonary TB: a direct sputum smear was positive on one or more occasion. Laboratory proven TB: smear positive and/or a positive *Mycobacterium tuberculosis* culture obtained from specimens as reported by the National Health Laboratory Services, Cape Town [7].

### HIV-status

Notified patients were defined as HIV-positive if results of positive HIV serology were recorded in the TB register or if the patient was recorded to be currently receiving antiretroviral therapy or co-trimoxazole prophylaxis. HIV-negative status was defined by a recorded HIV-negative serology result. All other cases were considered to be of unknown HIV-status.

### Cumulative risk analysis

Cumulative risk of TB was modeled using standard life table analysis [14]. In the absence of longitudinal data, age bands were used as surrogates for time. Age-specific TB notifications and adjusted denominators of population at risk for TB incidence in preceding age bands were used to calculate age specific TB rates. The cumulative risk at any age band was estimated from the product of risks of not being infected (1-adjusted notification rate) in all the preceding age bands. Cumulative analyses were performed using only the known HIV-negative rates and also including an allocation of HIV-unknown cases in the same proportion as occurring among the known HIV-tested cases. As this was a cross-sectional study these hypothetical cumulative estimates assume persistence of the current 2009 *status quo* throughout life [14].

### Statistical methods

Simple descriptive statistics were used to characterize and compare groups of patients using Wilcoxon rank-sum, t-test, and Chi Square tests as appropriate.

### Human Subjects Protection

Since this analysis was performed at population level with notified anonymous data, ethical review and informed consent was not obtained.

Cape Town 2009 notification data were reported in a different analysis by the same authors in an editorial [15].

## Results

### Cape Town Population

**Figure 1:**
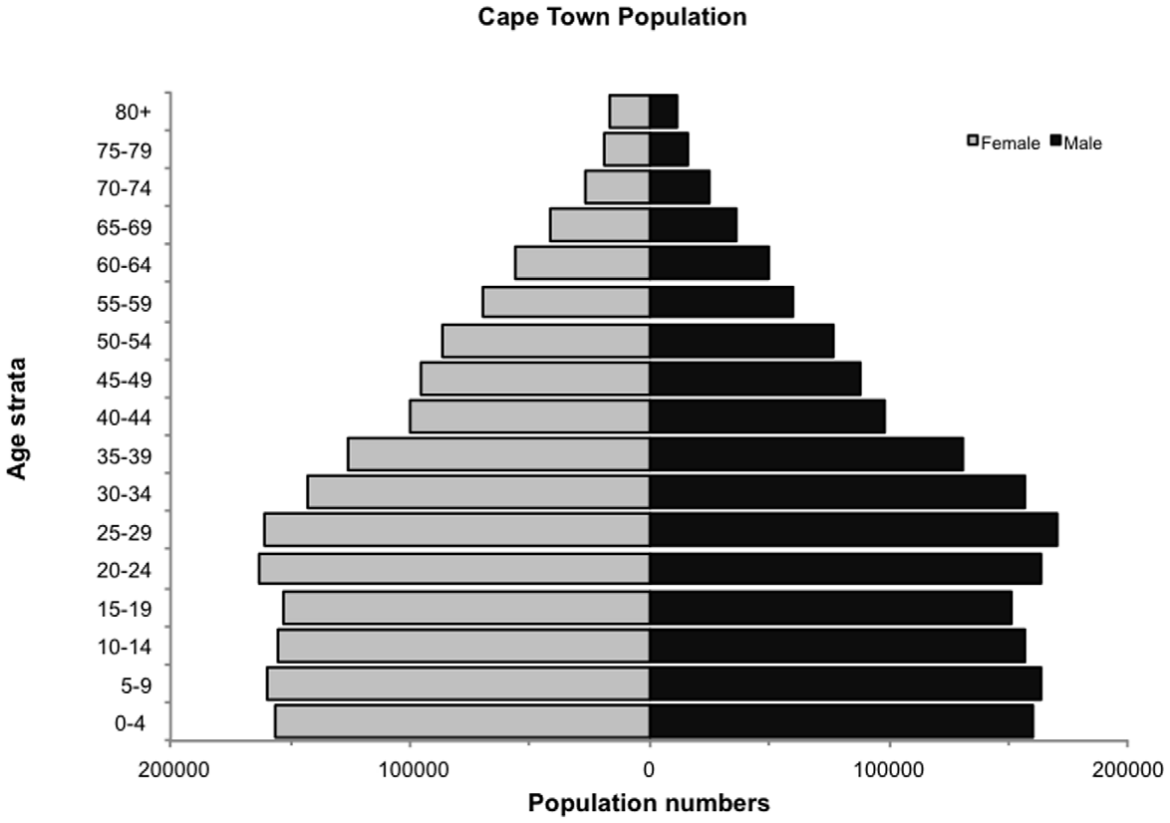
Population age and gender pyramid of Cape Town at mid-year 2009. Data based on South African census data [10].

The mid-2009 population of Cape Town was estimated to be 3,443,010 and the age and gender population pyramid for the population is shown in figure 1. Age-stratified denominators are given for overall population and for the HIV-uninfected and HIV-infected population pools (Table 1).

**Table 1:** The number of tuberculosis notifications (numerators) together with Cape Town population (denominators) stratified by both 5-year age groups and HIV-status.

	Cape Town population			TB HIV-negative			TB HIV-positive			TB HIV-status unknown		
Age strata	Total	HIV-	HIV+	All cases	New cases	Smear+	All cases	New cases	Smear+	All cases	New cases	Smear+
0–4	316078	309448	6630	1582	1559	7	311	291	6	960	948	1
5–9	322819	319326	3493	372	362	19	133	122	11	217	214	6
10–14	312137	311707	430	227	217	98	75	61	17	91	89	29
15–19	304903	301447	3456	988	887	617	197	173	71	251	238	150
20–24	327113	306210	20903	1693	1368	1143	1098	871	408	393	339	240
25–29	330742	287160	43582	1381	1030	906	2537	1909	858	376	307	246
30–34	298919	251725	47194	1015	721	673	2973	2060	986	296	230	166
35–39	257267	224038	33229	1042	657	682	2452	1607	832	268	172	155
40–45	197947	179524	18423	1053	628	662	1584	1006	545	203	133	120
45–49	183414	171395	12019	1076	593	656	954	615	318	215	132	132
50–54	163071	156238	6833	829	477	467	493	308	140	157	91	85
55–59	130157	126738	3419	571	345	320	269	171	62	123	84	65
60–64	106269	104718	1551	315	196	151	105	65	39	73	57	30
65–69	77547	77231	316	195	129	77	34	17	8	50	37	19
70–74	52142	52120	22	72	47	25	11	6	4	24	18	7
75+	62485	62485	0	97	74	37	6	5	3	41	30	12
Total	3443010	3241508	201502	12508	9290	6540	13232	9287	4308	3738	3119	1463

Tuberculosis case notification numbers for HIV-infected, HIV-uninfected and unknown HIV-status are also shown for new (first episode) and direct smear positive pulmonary tuberculosis.

### Tuberculosis notifications

**Figure 2:**
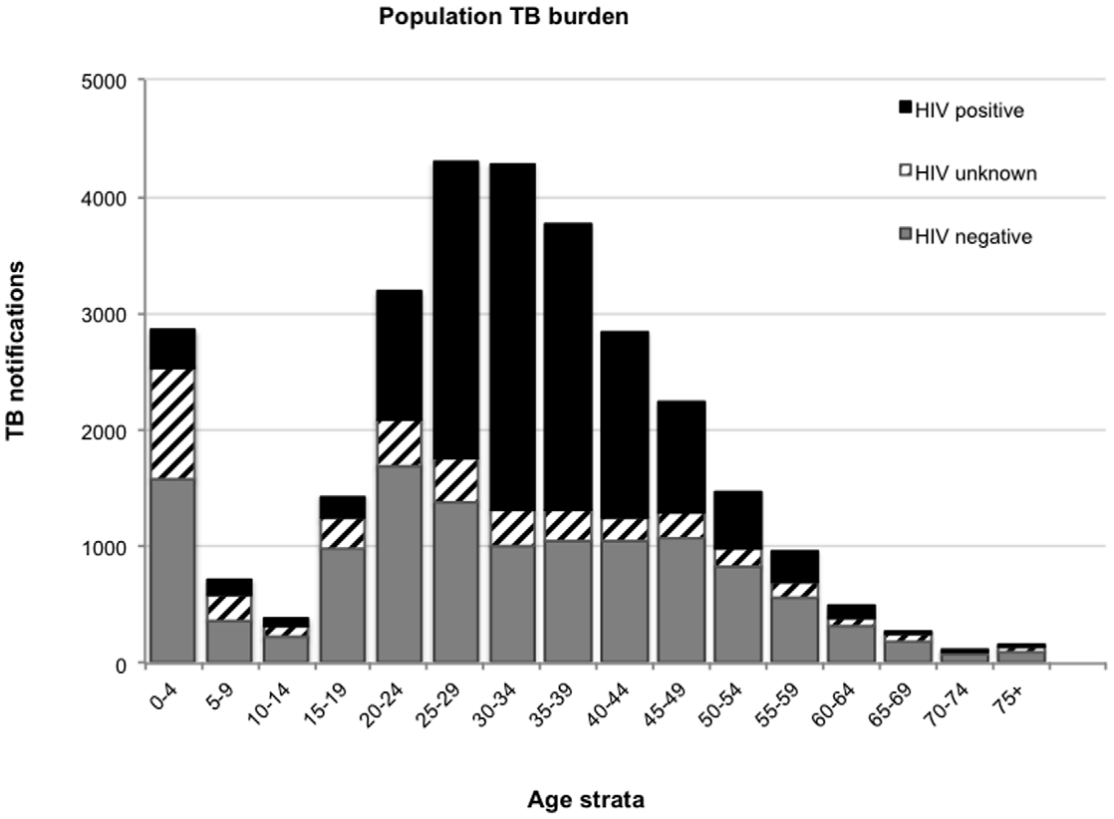
The numbers of tuberculosis notifications in 2009, which were recorded as HIV-infected, HIV-uninfected and of unknown HIV-status, stratified by 5-year age group.

In the Cape Town metropolitan area a total of 31,093TB cases were recorded in the electronic TB register of the TB control program between January 1^st^ and December 31^st^2009. “Transfers in” (1,331) and “moved in” (284) cases were excluded from the analysis. Of the remaining 29,478 newly registered TB cases, 12,311 (42%) were smear-positive pulmonary cases and of these, a further 4,175 (14%) confirmed by culture of *Mycobacterium tuberculosis*. The numbers of all TB notifications, new TB notifications and laboratory confirmed cases are show stratified by age in table 1. HIV status was available for 25,740 (87.3%) of whom 12,508(48.6%) were HIV-negative and 13,232 (51.4%) were HIV-positive. The number of TB notifications (TB burden), which were of HIV-negative, HIV-positive and unknown HIV-status are shown, stratified by age in figure 2. There were 2,853cases of childhood TB notifications in the 0–4 year's age-group and 1,115 between 5 and 14 years of age. Of the 25,510 adult cases, HIV-negative burden was greatest between age 20- and 24 years and HIV-positive between age 30- and 34 years.

### Effect of unknown HIV-status on TB rates

HIV-status was unknown in 13% of the total TB notifications, however testing rates varied considerably across age strata. The testing proportion was lowest (66%) among 0–4 year-olds, was highest (91.2%) in adults between 20 and 59 years and lower (82%) in those above 60 years. HIV-negative rates for 0–4 years may therefore be underestimated by 34% whereas adult (20–59 years) under ascertainment was likely less than 10%. For HIV-positives under ascertainment of numerators together with small denominator numbers at the extremes of age could combine to affect rate estimations in the very young and elderly. HIV-positive analyses were therefore restricted to ages 20 to 59 years where there was high ascertainment of numerators and well-characterized denominators.

### Non HIV-associated TB burden

**Figure 3:**
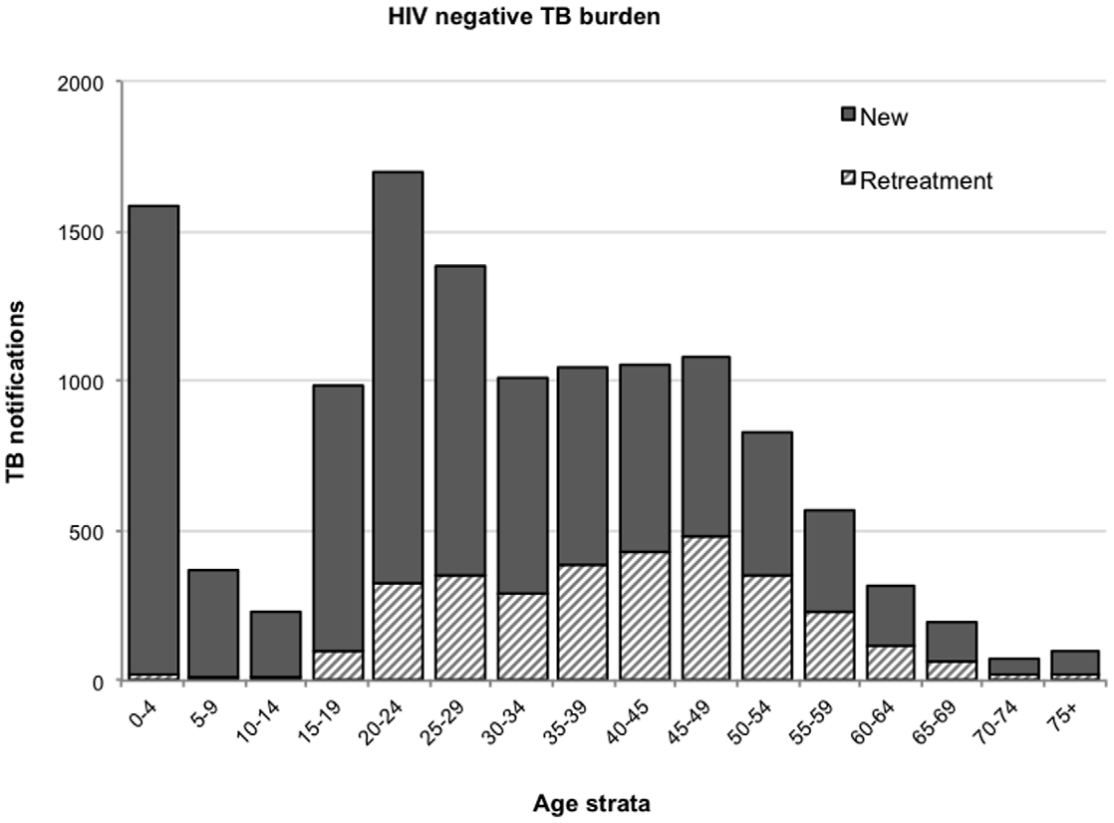
The numbers of tuberculosis notifications of new and recurrent disease in patients with known HIV-negative status, stratified by 5-year age group.

The burden of disease (TB notifications) for new and retreatment TB stratified by age is shown for HIV-negative individuals in figure 3. These estimates represent the minimum burden and include no allocation of cases from those with unknown HIV status. There were 1,582 cases between ages 0–4 years, with an age-stratified nadir of 227 cases between 10- and 14 years and higher TB rates seen with increasing adolescent age. Recurrent disease constituted 26% (n = 3,218) of the total burden of which 84.5% (n = 2,718) had no history of treatment failure or default.

### Non HIV-associated TB rates

**Figure 4:**
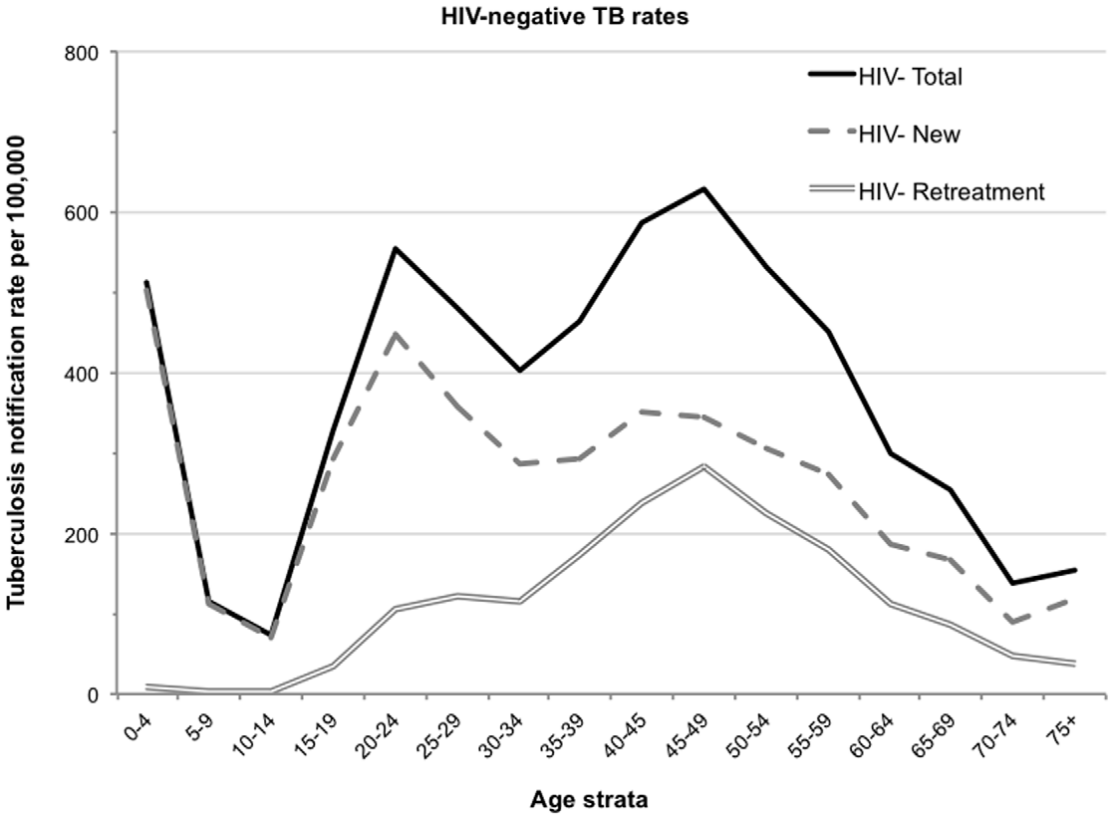
Notification rates for all, new and recurrent tuberculosis cases for HIV-negative residents aged 15 years to 49 years in Cape Town. HIV-uninfected population denominators derived from the product of Cape Town age population pyramid [10], [11] and 1-HIV-prevalence [12].

TB notification rates (cases per 100,000) calculated from the known HIV-negative new and retreatment TB cases and the estimated HIV-negative population of Cape Town are shown stratified by age in figure 4.There were 3 distinct peaks of TB incidence. The first peak was at 0–4 years of 511cases per 100,000 of which 1.5% were retreatment cases. Following the nadir between 10- and 14 years, TB rates increased rapidly with increasing adolescent age to a second peak of 553 cases per 100,000 of which 19.2% were due to retreatment TB. The highest TB incidence rate of 628/100,000 was among individuals aged 45–49 years in whom retreatment TB constituted 45% of incidence.

### Cumulative non HIV-associated TB risk

**Figure 5:**
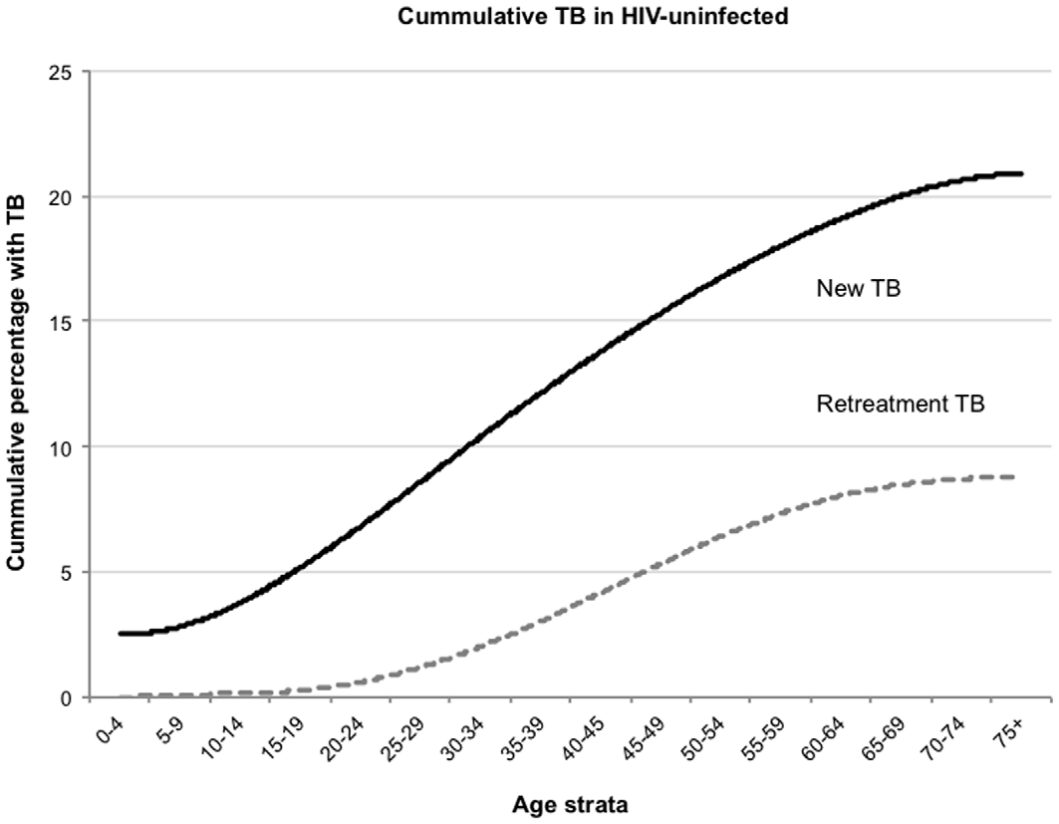
The estimated cumulative risk for new (initial) and retreatment episodes of tuberculosis between birth and 75 years of age for Cape Town residents remaining HIV-uninfected.

A modeled estimate of the lifetime risk for HIV-uninfected individuals of developing either an initial or retreatment episode of TB is shown in figure 5. The model calculates the hypothetical cumulative risk for individuals who remain HIV-uninfected and assumes current TB incidence rates persist throughout their life. Residents of Cape Town surviving to the age of 70 years and remaining HIV-uninfected would have a minimum 20% lifetime risk for having any new TB diagnosis and an estimated 24%after adjustment by allocation of unknown HIV-status cases. The corresponding lifetime risks for retreatment TB were a minimum of 8.4% and adjusted rate with unknown case allocation of 9.2%. The adjusted lifetime rate for smear-positive pulmonary TB was 12% for new TB and5.5% for retreatment TB. The cumulative risk for retreatment episodes was twice that expected by allocating equal risks to initial and subsequent episodes (initial risk squared), indicating that those with a primary treated TB event are at an approximate two-fold increased likelihood for developing a retreatment episode.

### HIV-infected TB burden

**Figure 6:**
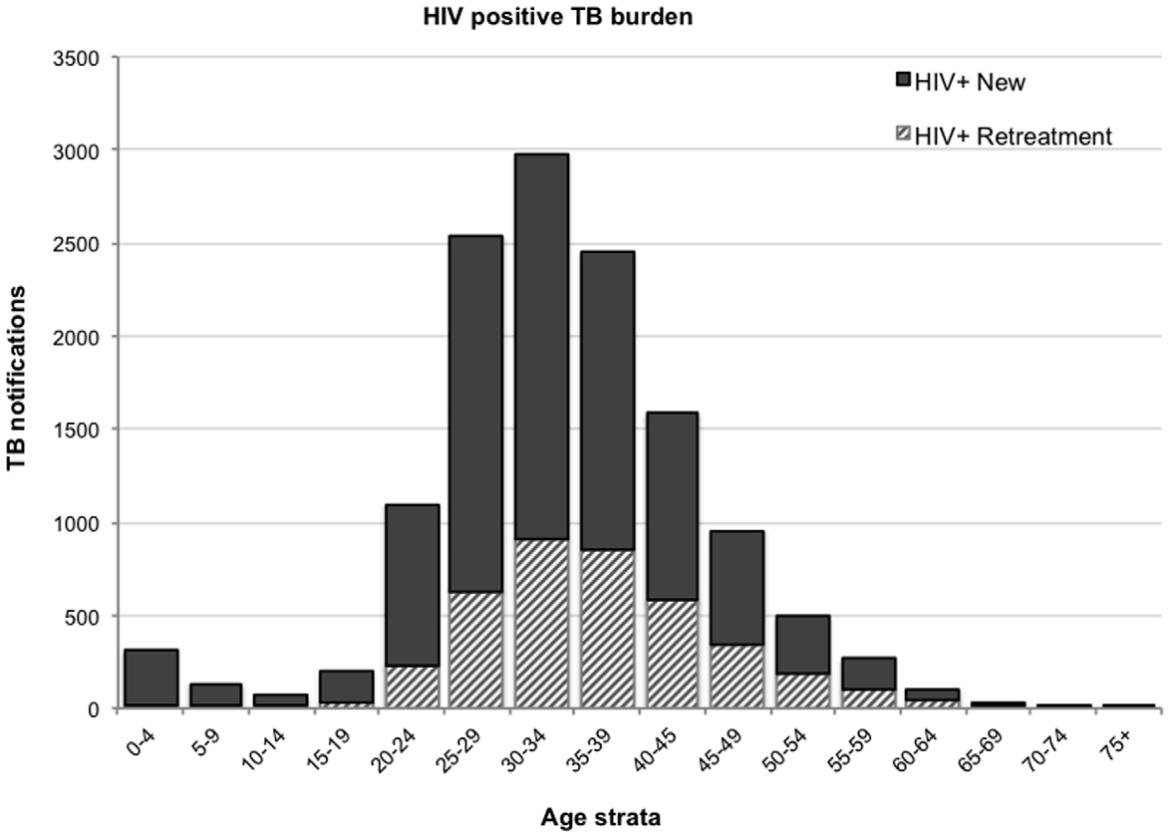
The numbers of tuberculosis notifications of new and recurrent disease in patients with known HIV-infection, stratified by 5-year age group.

The number of new and retreatment HIV-infected notifications stratified by age is shown in figure 6. These values represent the minimum burden and include no allocation of cases from those with unknown HIV status. Retreatment TB cases constituted 30% (n = 3,945) of the total burden of which87.6% (n = 3,457) had no history of treatment failure or default. Retreatment cases occurred at older age (35*vs* 32 years p<0.001) and had higher case fatality (6.4% *vs.* 5.1% p<0.01) than new TB cases. CD4 cell counts were available for 3,508 of 3,945 (89%) retreatment cases and 8,307 of 9,287 (89%) new cases. The median CD4 cell count of retreatment TB cases was higher (166 cells/uL *vs*148 cells/uL, p = 0.02) than that of new cases.

### HIV-infected TB and antiretroviral therapy

1,288 patients were recorded to be receiving ART at the time of TB notification, which represents 9.7% of total HIV-positive notifications. The median age of individuals on ART was a little older (34 years *vs.* 33 years, p = 0.052) and the median CD4 cell count moderately lower than non-ART cases (147cells/uL*vs* 153cells/uL, p = 0.058). However, the proportion of patients receiving ART in whom disease was due to retreatment was significantly higher compared to that of non-ART cases (43% versus 28%, p<0.001). The proportion of retreatment cases following default or failure was lower in the on-ART group (9.4%, 95%CI 7-11.83) compared with non-ART cases (12.6%, 95% CI 11.5-13.7).

### HIV-infected TB rates

**Figure 7:**
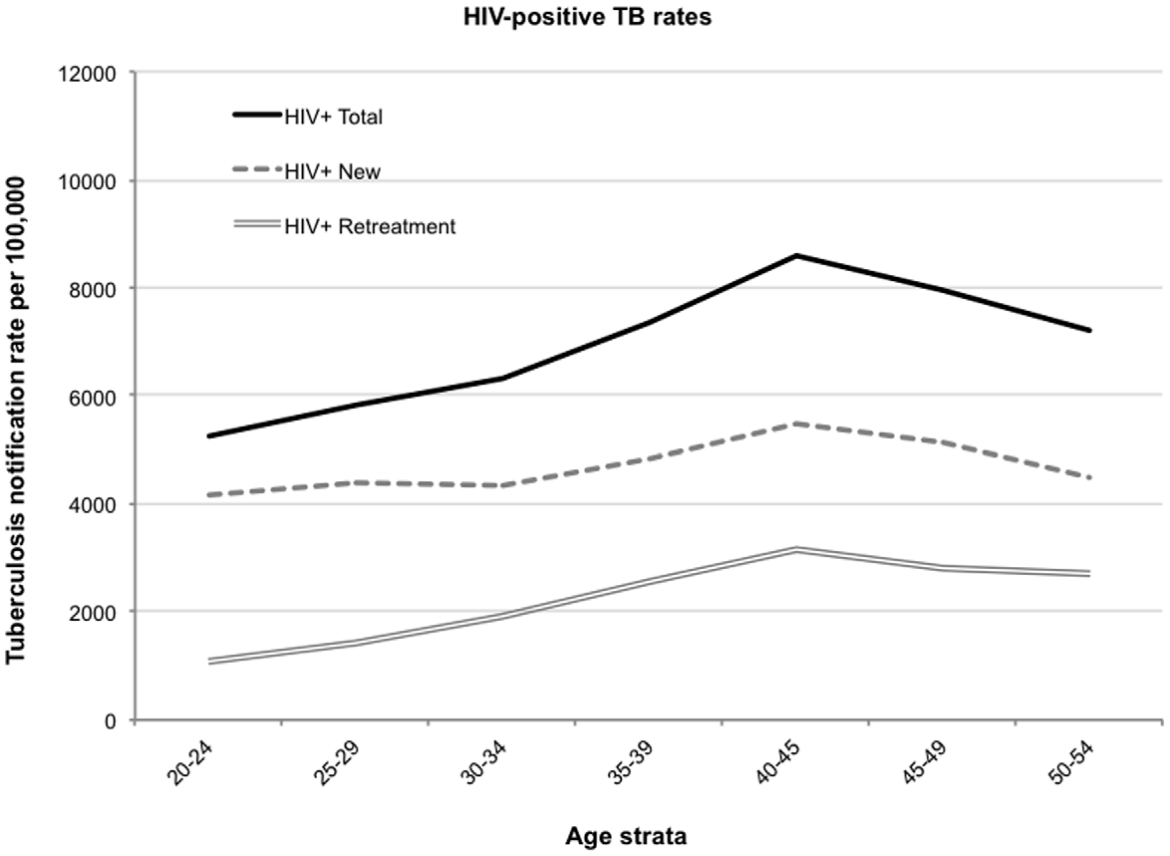
Notification rates for all, new and recurrent tuberculosis cases for HIV-positive residents aged 20 years to 54 years in Cape Town. HIV-infected denominators derived from the product of Cape Town age population pyramid [10], [11] and HIV-prevalence [13].

Minimum TB notification rates calculated from the known HIV-positive new and retreatment TB cases and the estimated HIV-positive population of Cape Town are shown by 5-year age strata between 20–24 years and 50–54 years in figure 7. The new HIV/TB notification rate remained relatively stable with increasing age (4,392 to 4,561 cases/100,000) whereas recurrent disease rates increased markedly with age from 1,092 at 20–24 years to 2,265 cases/100,000 at 50–54 years.

## Discussion

For the first time in a major city in sub-Saharan Africa with high HIV and TB burden, a very high rate of HIV testing of TB cases has permitted careful analysis of the burden of HIV-associated and non-HIV-associated TB stratified by sex and age. The TB notifications from this city were more than twice the combined annual caseload of USA and Canada [1] and rates of both HIV-associated and non-HIV-associated TB was extremely high. TB incidence rates in HIV-negative were greatest in 45–49 year olds and very high in the 20–24year olds, and were of similar magnitude as those reported in the 1940's in Norway [16]. The estimated lifetime TB risk of 22% was approximately double that observed in studies of TB infection acquired during childhood in United Kingdom in the 1950's [17], [18] and was similar to estimates of cumulative TB risk in the early 20^th^ century Europe prior to advent of chemotherapy [19].

An important finding in this study was that the proportion of the total TB burden due to retreatment disease (26%) was greater than that reported for South Africa (18.8%) or the African continent (9.9%) [1], [20]. Retreatment cases may result from failed initial therapy or subsequent reactivation or re-infection [21]–[24]. The proportion of cases reported after treatment failure or default reflects the cases due to failed initial treatment. Despite an increased burden of retreatment TB cases in Cape Town the proportion following failed initial therapy (14.3%) was lower than that reported for South Africa as a whole (15.7%) or for the African continent (22%) [1]. Furthermore, HIV-positive cases had a higher retreatment rate than HIV-negatives but a lower proportion due to failed prior treatment. Together these data suggest that the high retreatment rate in Cape Town is unlikely to be predominantly due to inadequate or poor case management but is more likely related to the high prevailing force of TB infection [25]–[28]. The high cumulative risk for recurrent disease in HIV-negatives also indicated that following an initial TB episode there was a two-fold increased risk for subsequent TB episodes. This increased hazard for subsequent TB episodes following an initial TB episode confirms previous reports from districts within Cape Town [29]. Speculative reasons for this increased risk may include that these individuals have higher environmental exposure, (i.e., more TB contacts) or that latent infection itself may offer some immunologic protection and treatment may interfere with this immunity.

Non-HIV associated TB varied markedly between age strata and 3 peaks in TB incidence were observed. The first peak occurred before 4 years of age. HIV testing was low in infancy and the therefore even the high HIV-uninfected childhood's notification rate may have been underestimated by as much as 33%. This TB disease rate is in keeping with a reported annual risk of TB infection (ARTI) of approximately 4% per annum in young children in Cape Town [25], [26], which has remained largely unchanged over the last decade [30]. The ARTI in poor communities of Cape Town TB is maintained by transmission to young children within households from adults with smear-positive pulmonary disease [27], [31]. Despite limited social networks, young children have prolonged contact in poorly ventilated dwellings with extended family members and frequently share sleeping quarters with adults [27].

Childhood TB notifications rapidly decreased after the age of 5 years to a nadir between 10 and 14 years. This decline in TB disease occurred despite a high continuing annual TB infection rate [25]-[28], a phenomenon that has been widely recognized but is poorly understood [21]. Whilst it may be speculated that this has an immunologic aetiology, further research to understand this phenomenon is warranted. TB notification rates rapidly increased from the nadir at 10–14 years to a second peak between 20 and 24 years. As TB disease is more frequent soon after infection [16]–[19], [21] this rapidly increasing incidence is consistent with very high infection rates (7% per annum) reported among adolescents in Cape Town [28].

The third peak in TB notification rates occurred at age 45–49 years and consisted of almost equal proportions of new and recurrent TB disease. Recurrent disease in Cape Town and other high burdened settings has been reported to result predominantly from re-infection [22]–[24]. Multiple re-infections are to be predicted when the prevailing force of TB infection exceeds 1% per annum [32], [33].

The high lifetime risk for developing infectious pulmonary disease has serious implications for TB control. An effective TB contact number is defined as a contact between an infectious pulmonary cases and a susceptible individual sufficient to result in TB infection [34]. In this population where 1 in 8 of the HIV-negative population develops infectious smear-positive pulmonary TB, the effective contact number should be reduced to less than 12 for long-term epidemic control within the HIV-negative population. Historically the effective contact number in the United Kingdom was estimated to decline from 22 in 1900, to 10 in 1950 and 1 in 1990 [19].

HIV-infected individuals had a 17-fold increased risk of TB compared with HIV-negative peers and the burden of HIV-positive TB closely mirrored the prevalence of HIV-infection in the city. Interpretation of age specific incidence is more complex in the HIV-positive population as age is only indirectly related to time from acquisition of HIV-infection and access to ART is an increasing confounder. However, there were indications that the rate of new and retreatment TB may be differentially affected by HIV-infection. Whilst the reasons for this are as yet speculative, the younger age of new HIV/TB cases together with a lower CD4 cell count may be an indication that some new case TB may be a manifestation of a transient CD4 decline which occurs following sero-conversion. In contrast, the older age and higher rate of recurrent disease with age may reflect a combination of high ongoing risk of TB infection and HIV disease progression. Increased retreatment TB among those on ART may also indicate that re-infection TB is particularly common in this group. In addition, as evidenced by higher CD4 T cell counts in the retreatment group on ART, this may reflect longer survival in an ongoing risk of TB reinfection.

Weaknesses of this study include the analysis of routinely collected data within a busy TB control program with the consequential risk of miss-classification of patient category status. Furthermore, there may also have been unascertained TB cases, which died before notification to the TB control program. The denominator estimations were based on an actuarial model of the South African HIV epidemic (ASSA model) and was not directly measured. However, the ASSA model is calibrated against the South African antenatal sero-prevalence surveys performed annually. The ASSA model has been widely used for health system planning including by the South African department of health and treasury. Cumulative risk analyses were based on data from a single year and can only reflect an estimation assuming continuation of the *status quo*. The HIV-testing proportion of 87% is still not optimal but is considerably higher than national testing rate and that of any other comparable large population. Whilst reported rates of multidrug resistant TB prevalence is estimated to be about 2% in new TB patients and 7% in re-treatment cases [35], drug susceptibility testing is not routinely performed according to the National TB program and therefore the role that drug resistance may play in treatment failure and retreatment was not possible to evaluate in this study. The strength of the study is the large number of cases, analyzed within a well functioning TB control program with high quality laboratory support.

In conclusion, HIV-infection undoubtedly contributes significantly to the increased TB caseload noted in recent years. However, while much of the public health focus has been understandably directed towards HIV-associated TB, non-HIV associated TB incidence rates have been underestimated and we report TB burdens now similar to those recorded in the pre-chemotherapy era. The high retreatment rates observed in both HIV-positive and HIV-negative populations do not appear to be due to failed initial TB therapy but these data are indicative of a city population that is subjected to an extremely high and ongoing force of TB infection. In addition to effective TB case management, it will be necessary to revisit those TB control measures, which effectively reduced TB transmission in industrialized countries before the discovery of TB chemotherapy.
